# Equivalence of multibreed animal models and hierarchical Bayes analysis for maternally influenced traits

**DOI:** 10.1186/1297-9686-42-20

**Published:** 2010-06-11

**Authors:** Sebastián Munilla Leguizamón, Rodolfo JC Cantet

**Affiliations:** 1 Departamento de Producción Animal, Facultad de Agronomía, Universidad de Buenos Aires, Buenos Aires, Argentina; 2 Consejo Nacional de Investigaciones Científicas y Técnicas, Argentina

## Abstract

**Background:**

It has been argued that multibreed animal models should include a heterogeneous covariance structure. However, the estimation of the (co)variance components is not an easy task, because these parameters can not be factored out from the inverse of the additive genetic covariance matrix. An alternative model, based on the decomposition of the genetic covariance matrix by source of variability, provides a much simpler formulation. In this study, we formalize the equivalence between this alternative model and the one derived from the quantitative genetic theory. Further, we extend the model to include maternal effects and, in order to estimate the (co)variance components, we describe a hierarchical Bayes implementation. Finally, we implement the model to weaning weight data from an Angus × Hereford crossbred experiment.

**Methods:**

Our argument is based on redefining the vectors of breeding values by breed origin such that they do not include individuals with null contributions. Next, we define matrices that retrieve the null-row and the null-column pattern and, by means of appropriate algebraic operations, we demonstrate the equivalence. The extension to include maternal effects and the estimation of the (co)variance components through the hierarchical Bayes analysis are then straightforward. A FORTRAN 90 Gibbs sampler was specifically programmed and executed to estimate the (co)variance components of the Angus × Hereford population.

**Results:**

In general, genetic (co)variance components showed marginal posterior densities with a high degree of symmetry, except for the segregation components. Angus and Hereford breeds contributed with 50.26% and 41.73% of the total direct additive variance, and with 23.59% and 59.65% of the total maternal additive variance. In turn, the contribution of the segregation variance was not significant in either case, which suggests that the allelic frequencies in the two parental breeds were similar.

**Conclusion:**

The multibreed maternal animal model introduced in this study simplifies the problem of estimating (co)variance components in the framework of a hierarchical Bayes analysis. Using this approach, we obtained for the first time estimates of the full set of genetic (co)variance components. It would be interesting to assess the performance of the procedure with field data, especially when interbreed information is limited.

## Background

Mixed linear models used to fit phenotypic records taken on animals with diverse breed composition are termed multibreed animal models. Theoretical [1,2] and empirical [3,4] arguments indicate that the proper specification for the genetic covariance structure in these models should be heterogeneous. However, even though the theory has long been developed [1,5,6] and classical [3,7] and Bayesian [4] inference procedures have been presented, very recent papers on (co)variance component estimation in crossbred populations (e.g., [8,9]) do not account for this particular dispersion structure, possibly due to the lack of appropriate general purpose software [10].

Estimation of (co)variance components in multibreed populations is not an easy task [3,4,11]. Basically, the difficulty arises because the scalar (co)variance components can not be factored out from the inverse of the additive genetic covariance matrix. As a consequence, within the framework of a hierarchical Bayes analysis the full conditional posterior distribution of each (co)variance component is not recognizable, and thus algorithms such as Metropolis-Hastings must be used [4].

The approach based on the decomposition of the genetic covariance matrix by source of variability [10] supplies a much simpler formulation for (co)variance component estimation, which is easy to assimilate with the collection of estimation techniques available in general purpose software. García-Cortés and Toro [10] have empirically illustrated the validity of their proposal through a numerical example, but they have not presented a formal derivation of the equivalence between their model and the one formalized by Cantet and Fernando [2] using the quantitative genetic arguments of Lo et al. [1], at least when the goal is to predict breeding values.

In this study we address the issue. Basically, we will present a formal derivation of the equivalence through a somewhat different formulation from the one of García-Cortés and Toro [10]. Further, we will expand the model to include maternal effects, and formalize a hierarchical Bayes analysis to estimate the parameters of interest. Finally, the multibreed analysis discussed above is used in the analysis of weaning weight records from an Angus × Hereford crossbred experiment.

## Methods

### Equivalence of multibreed animal models

For the sake of simplicity, assume a two-breed (*A *and *B*) composite population with individuals pertaining either to one of the two parental breeds, or to one of several breed groups produced by crossbreeding. The trait of interest is under the influence of a large number of unlinked loci, and the two parental breeds that give rise to the population are in gametic phase equilibrium. Thus, assuming additive inheritance, the genotypic value of individual *i *in any breed group can be modeled as(1)

where μ is the mean genotypic value in the reference breed group, and ,  represent, respectively, the additive effects of the paternal and maternal alleles that individual *i *inherited at locus *t *(*t *= 1, ..., *n*). In this context, Lo et al. [1] have derived the expression for the variance of the genotypic value as a linear function of the additive variance in each parental population, and an additional source of variability arising due to differences in allelic frequencies between these populations: the segregation variance [12,13]. In the two-breed case, it is equal to(2)

where  and  respectively are the expected proportion of breed *A *and breed *B *genes in individual *i*,  and  are the additive variances of each breed, and  is the segregation variance. The last term in (2) stands for the covariance between genotypic values for the parents of the individual, and can be developed further by expanding to the previous generation. Under this formulation, Lo et al. [1] have shown how to compute efficiently both the genetic covariance matrix using the tabular method [14], and its inverse using the algorithms of Henderson [15] and Quaas [16]. Later, Cantet and Fernando [2] have demonstrated how to use the theory to predict breeding values by BLUP within the framework of a genetic evaluation.

Alternatively, García-Cortés and Toro [10] have decomposed the genetic covariance matrix into several independent sources of variability. In the two-breed situation it is verifiable that(3)

where ***A***_*X*_, *X* = {*A*, *B*, *S*}, are partial numerator relationship matrices in accordance with the source of variability [10]. These matrices have order *q *× *q *(where *q *is the number of individuals) to ensure conformability for addition. However, if an individual does not contribute to the source of variability (for example, purebred *A *individuals does not contribute to *B *and *S *sources of variation) the corresponding row and column are null vectors, and thus the matrix is singular. This formulation of the genetic covariance matrix is consistent with a conventional animal model with several random effects, i.e., the breeding values by breed origin , ***a***_*X*_, *X* = {*A*, *B*, *S*}. It should be clear that under this alternative model the breeding values of non-contributing individuals to a particular source of variability are defined to be fixed and equal to zero, and are termed null by breed origin.

The alternative formulation presented by García-Cortés and Toro [10] alleviate difficulties inherent to (co)variance components estimation within multibreed animal models, specially through estimation techniques based in known full conditional distributions (i.e., Gibbs Sampler), within the framework of a hierarchical Bayes analysis. Furthermore, the referred model is equivalent to the model presented by Cantet and Fernando [2] in terms of the covariance structure, because both formulations are identical (see the definition given by Henderson [17]). Yet, the equivalence in terms of breeding value prediction is not straightforward, because the coefficient matrix derived form the mixed model equations is singular, and equations corresponding to non-contributing individuals have to be discarded in order to solve the system and to obtain equivalent results [10].

Our proposal is to redefine the ***a***_*X*_ vectors such that they only include the *q*_*X*_ breeding values non-null by breed origin. This entails defining appropriate incidence matrices ***Z***_*X*_ for each source and rewriting the model equation as(4)

where ***Z***_*X*_ of order *n *× *q*_*X *_are related to the *q*_*X *_non-null breeding values by breed origin , *X* = {*A*, *B*, *S*}. Note that this formulation does not include breeding values constrained to zero, so that , where the non-singular matrix  contains the non-null rows and columns of ***A***_*X*_. Define then the matrix ***M***_*X*_ of order *q *× *q*_*X*_, such that(5)

where ***Z*** is the incidence matrix for the random effects in [10] and [2]. It is then verifiable that the product  retrieves the null-row pattern with respect to matrix ***A***_*X*_. In turn, a subsequent post-multiplication by , retrieves the null-column pattern, so that(6)

Using (6) and (5) in (4)(7)

This result shows that model (4) is equivalent to the model presented by Cantet and Fernando [2] in accordance to the definition given by Henderson [17]. Moreover, note that the BLUP of each non-null breeding value by breed of origin can be written according to [18](8)

Now, both expressions (6) and (8) can be used to show that the addition of the , weighted by the corresponding ***M***_*X*_ matrices to ensure conformability, equals(9)

where  = *BLUP*(***a***) from the multibreed animal model presented by Cantet and Fernando [2]. Finally, note that even though we have assumed a two-breed composite population in our presentation, the argument readily generalizes to a multibreed population composed of *p *breeds.

### Hierarchical Bayes analysis for a maternal multibreed animal model

Consider now a maternally influenced trait, and assume therefore the covariance structure described by Willham [19]. Additionally, consider the theory of Lo et al. [1] extended to correlated traits as presented by Cantet and Fernando [2]. We will use subscripts "*o*" and "*m*" to differentiate between direct and maternal effects, respectively. Then, using the approach presented in the previous section, we define the model(10)

where ***y*** (*n *× 1) is a data vector, and ***X*** (*n *× *p*) represents, without loss of generality, the full-rank incidence matrix of the fixed effects vector ***b*** (*p *× 1). Furthermore,  and  are random vectors with entries corresponding to the *q*_*X *_direct and maternal non-null breeding values by breed origin *X*, *X* = {*A*, *B*, *S*}. Note, respectively, and ***e***_*p*_ (*d *× 1) is a random vector accounting for maternal permanent environmental effects. Accordingly, ***Z***_*oX*_, ***Z***_*mX*_ and ***Z***_*p*_ are the corresponding incidence matrices. Finally, ***e***_*o*_ (*n *× 1) represents the white-noise error vector. To simplify the notation, let ***Z***_*X*_ = [***Z***_*oX*_ | ***Z***_*mX*_] and .

Next, consider a hierarchical Bayes construction for model (10) as presented by Cardoso and Tempelman [4] following Sorensen and Gianola [20]. The objective is to make inferences about parameters of interest, typically the (co)variance components. At the first stage of the analysis, it is necessary to specify the full conditional sampling density of the data vector. Assume therein a multivariate normal process(11)

Then, the prior distributions for vectors ***b***, , *X* = {*A*, *B*, *S*}, and ***e***_*p*_ are specified. Firstly, a multivariate normal process will be assumed for the vector of fixed effects ***b***. This assumption avoids the occurrence of improper posterior distributions, while reflecting a prior state of uncertainty for the fixed effects [21]. According to Cantet et al. [22], we set(12)

where ***K*** = *Diag*{*k*_*i*_}, with *k*_*i*_ ≥ 1 × 10^7^ for *i *= 1, ..., *p*.

Secondly, multivariate normal distributions will also be specified for the non-null breeding values by breed origin , according to quantitative genetic theory(13)

In (13),  and  represents the partial numerator relationship matrices defined by García-Cortés and Toro [10], but without null rows and columns. Finally, a multivariate normal process will be assumed for the vector of maternal permanent environmental effects. Thus(14)

In the next level of the hierarchy, a priori distributions are to be assigned to the dispersion parameters, i.e., the scalars  and , and the matrices ***G***_0*X*_, *X* = {*A*, *B*, *S*}. At this point, conjugate scaled inverted-gamma densities are assumed: Inverted Chi-squared for the scalars and Inverted Wishart for the matrices. Then(15)

In (15),  are (2 × 2) matrices containing the a priori values for the genetic (co)variance components for each source of variability. Moreover,  and  represent prior values for the maternal permanent environmental variance and the white-noise error variance, respectively. All these values should be interpreted as statements about the expectation of the prior distributions, and are defined by the analyst. In turn, υ_*X*_,  and  represent the parameters for the degrees of freedom of the corresponding distributions, and are interpreted as a degree of belief in those a priori values [20]. They are also defined by the analyst.

Now, assuming that ***b***,  |***G***_0*X*_, ***G***_0*X*_, *X* = {*A*, *B*, *S*}, ***e***_*p*_|,   and  are all a priori independent, the joint posterior distribution will be proportional to the product of the likelihood function times each of the prior densities, as follows(16)

Explicitly, and after grouping together common factors [20], we obtain(17)

where  and .

Starting with expression (17), it is possible to identify the kernel of the full conditional posterior density of any parameter of interest by keeping the remaining ones fixed. In fact, it is verifiable that all full conditional posterior densities are analytically recognizable and thus can be sampled using standard procedures as those described by Wang et al. [23] or Jensen et al. [24]. Detailed expressions for the full conditional posterior densities are derived and displayed in the appendix.

### Analysis of experimental data

In this section we describe the implementation of the hierarchical Bayes analysis to a data set from an Angus × Hereford crossbred experiment. Data belongs to the AgResearch Crown Research Institute, New Zealand, and consists of 3749 weaning weight records and the corresponding genealogy (Table 1). Records were collected between 1973 and 1990 on both purebred and crossbred individuals, including progeny from inter-se matings, backcrosses, and rotational crosses (Table 2). A detailed description of the mating design and other relevant features from the experiment can be found in Morris et al. [25].

**Table 1:** Characteristics of the pedigree and data file of the Angus × Hereford crossbred experiment

	ANGUS × HEREFORD		
**PEDIGREE file**	**Individuals**	**Bulls**	**Cows**

	4668	292	1698

**DATA file**	**N**	**Mean, kg**	**SD, kg**
			

WW records	3749	153.56	29.94
	
	**Sires**	**Dams**	**Total**
	
Parents	216	1647	1863
(with WW record)	145	923	1068
	
%	67.13	56.04	57.33
			
Mean number of calves by parent	16.05	2.28	

% of parents with:			
1 calf	3.70	42.93	
2 calves	4.17	21.86	
3 calves	2.31	15.66	
>3 calves	89.81	19.55	

WW = weaning weight; N = number of records; SD = standard deviation

Description of the data set used in the multibreed analysis, including several useful features for evaluating data quality for the estimation of (co)variance components within maternal animal models

**Table 2:** Mating types, genotypes and breed compositions represented in the Angus × Hereford data set

Mating type	Genotypes	N			
Parental	ANGUS	711	1.00	1.00	1.00
Parental	HEREFORD	431	0.00	0.00	0.00

Inter-se	F_1_(H × A)	393	0.50	0.00	1.00
Inter-se	F_1_(A × H)	301	0.50	1.00	0.00
Inter-se	F_2_(HA × HA)	235	0.50	0.50	0.50
Inter-se	F_2_(AH × AH)	183	0.50	0.50	0.50
Inter-se	F_3_(F_2_ × F_2_)	254	0.50	0.50	0.50
Inter-se	F_4_(F_3_ × F_3_)	104	0.50	0.50	0.50

Back-cross	B_1_(A × HA)	78	0.75	1.00	0.50
Back-cross	B_1_(A × AH)	72	0.75	1.00	0.50
Back-cross	B_1_(H × HA)	77	0.25	0.00	0.50
Back-cross	B_1_(H × AH)	67	0.25	0.00	0.50
Back-cross	B_1_(AH × A)	180	0.75	0.50	1.00
Back-cross	B_1_(HAxH)	132	0.25	0.50	0.00

Rotational	R_3_[A × B_1_(H × HA)]	77	0.63	1.00	0.25
Rotational	R_3_[A × B_1_(H × AH)]	51	0.63	1.00	0.25
Rotational	R_3_[H × B_1_(A × HA)]	96	0.38	0.00	0.75
Rotational	R_3_[H × B_1_(AH × A)]	51	0.38	0.00	0.75
Rotational	R_4_(A × R_3_)	67	0.69	1.00	0.38
Rotational	R_4_(H × R_3_)	68	0.31	0.00	0.63

Advanced	F_3_ × F_1_(HA)	19	0.50	0.50	0.50
Advanced	F_3_ × F_1_(AH)	27	0.50	0.50	0.50
Advanced	F_3_ × F_4_	30	0.50	0.50	0.50
Advanced	A × R_4_	21	0.66	1.00	0.31
Advanced	H × R_4_	24	0.34	0.00	0.69

**TOTAL**		**3749**			

, , : individual, sire and dam expected proportion of Angus genes (breed composition)

Mating types and genotypes are described in Morris et al. [25]; breed compositions are key features within the multibreed analysis: they are used both for computing the inverses of the partial numerator relationship matrices and as regressor variables for fitting the mean effects of breed groups

Our goal was to estimate (co)variance components inherent to this experimental population, thus we fitted the model presented in the previous section. The model included the non-null direct and maternal breeding values by breed origin, and fixed effects for sex, age of dam, and day of birth (fitted as a covariate), following the description given by Morris et al. [25]. To account for differences in the mean phenotypes between the breed groups, fixed effects of direct and maternal breed and heterosis were also included using the parameterization given by Hill [26,27].

(Co)variance components were estimated through a single-site, systematic scan Gibbs sampling algorithm, like the one suggested by García-Cortés and Toro [10]. The computation strategy in the current research was also based on setting-up the mixed model equations for an animal model with several random effects. However, instead of discarding equations corresponding to non-contributing individuals, these were never set up: the system was simply collapsed by changing the appropriate coordinates, i.e., by removing null rows and null columns. Note that this strategy has the advantage of reducing the number of necessary contributions, but it requires that all the animals with null contributions to any source of variability be identified.

Specifically, a FORTRAN 90 program was written, inspired on the class notes from Misztal [28]. The code is based on programs from the BLUPF90 package [29] and specific F77 routines from our research group [R.J.C. Cantet and A.N. Birchmeier, personal communication]. The program has a modular structure with two main internal subroutines. The first one generates the contributions to the random effects and computes the entries in the partial numerator relationship matrices according to a slightly modified version of the inbreeding algorithm of Meuwissen and Luo [30]. The second subroutine is used for sampling successively the vector of unknowns without setting-up the mixed model equations, thus accelerating considerably the performance by iteration. The code is available under request from the first author.

The implementation of the Gibbs sampling was undertaken in two stages. In the first stage, an exploratory analysis was done by seeking some reasonable values for the scale parameters of the prior distributions of the (co)variance components. First, a maternal animal model was fitted [19,31], and (co)variance components were estimated using the ASReml [32] package. Scale parameters for maternal permanent environmental and error variances densities were then set according to the REML estimates. Second, estimates of the genetic (co)variance components were arbitrarily distributed among the three sources of variability. Once prior values were chosen, the program was executed and several chains in between one and two million iterations were calculated, depending on the sign of the direct-maternal genetic covariances, the degrees of belief assigned to the parameters, and the number of samples discarded as burn-in. Posterior summaries and convergence diagnostics were reasonably consistent among all chains so that results are not shown. Finally, mean posterior mode values, taken among all the chains, were used to set the scale parameters of the prior distributions of the (co)variance components in the definitive analysis.

Based on these preliminary analyses, a large chain of 3,500,000 iterations was obtained in the second stage, following the suggestion of Geyer [33]. The first 100,000 samples were discarded as burn-in, and the remaining 3,400,000 were used to study convergence through all single-chain diagnostics supplied by the BOA [34] package, executed under the R [35] environment. Posterior means, modes, medians and standard deviations for all (co)variance components, as well as 95% high posterior density intervals (HPD), were computed using the program POSTGIBBSF90, from the BLUPF90 [29] package.

## Results

Relevant features regarding the implementation of the multibreed analysis to the Angus × Hereford data set are described below. The final analysis took about five days of execution on a personal computer with a Pentium^®^ 4 (CPU 3.6 GHz, 3.11 GB of RAM) processor, at a rate of 0.11 second per cycle. The numerical values used to initialize the scale parameters and the degrees of belief for the prior distributions of all (co)variance components are displayed in Table 3. Overall, auto-correlations among samples of the same parameter were very large for all (co)variance components, especially for those associated with the segregation terms. However, by using an appropriate thinning the auto-correlations decreased to reasonable values without affecting posterior summaries and, as a consequence, convergence was analyzed for the full length chain of 3,400,000 iterations. It is worth emphasizing that the sample sequences of all the (co)variance components succeeded in passing all single-chain convergence tests supplied by the BOA [34] package.

**Table 3:** Parameters a priori and posterior summaries for the marginal density of each (co)variance component

							HPD95	
							
CVC^1^		*S*^(0)^	Mean	SD	Median	Mode	Lower	Upper
	100	170	187.34	10.21	187.35	187.09	167.17	207.22
	100	80	95.53	9.91	95.24	98.75	76.47	115.17
	20	85	120.74	20.43	119.54	115.82	82.22	161.46
	20	-25	-27.00	13.26	-26.11	-23.89	-53.70	-2.15
	20	35	37.63	11.35	35.94	32.35	18.25	60.38
	20	76	100.24	20.12	98.86	98.42	62.38	140.33
	20	-50	-56.31	19.64	-55.12	-56.55	-95.65	-19.13
	20	70	95.18	24.61	92.96	88.29	50.29	144.21
	5	10	9.62	6.24	8.10	3.68	1.28	21.96
	5	8	9.55	7.01	7.82	3.20	0.36	24.18
	5	9	13.37	12.55	9.48	3.65	1.03	37.93

^1^(Co)variance components:  = error variance;  = maternal permanent environmental variance;  = direct additive variance by genetic origin;  = maternal additive variance by genetic origin,  = direct-maternal genetic covariance by genetic origin; *X *= {Angus, Hereford, segregation};  = a priori degrees of belief; *S*^(0)^ = a priori scale parameter; SD = standard deviation; HPD95 = 95% high posterior density interval.

**Figure 1:**
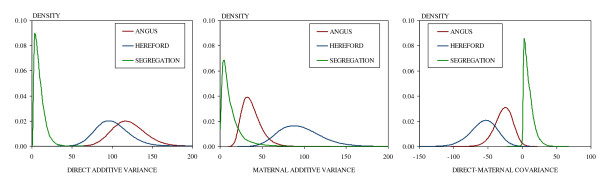
**Estimated marginal posterior densities for genetic (co)variance components disaggregated by breed source of variability**.

Table 3 displays the marginal posterior summaries for the eleven scalar (co)variance components of the fitted model. Additionally, Figure 1 displays the corresponding density shapes that were estimated using a non-parametric technique based on a Gaussian kernel [36]. In general, genetic (co)variance components showed marginal posterior densities with high degree of symmetry, except for those components associated with the segregation between breeds. In particular, while the mean values of direct and maternal segregation variances were respectively  = 9.62 kg^2^ and  = 13.37 kg^2^, the modes for both direct and maternal segregation variances were about 3 Kg^2^.

Besides, there were differences in the posterior summaries of the genetic (co)variance components according to the source of variability. First, there was a small scale deviation in the means of the direct additive variances between Angus and Hereford breeds:  = 120.74 kg^2^ vs.  = 100.24 kg^2^, respectively, both breeds having similar standard deviations. By contrast, the means of the maternal additive variances showed quite a large difference towards Hereford ( = 37.63 kg^2^ vs.  = 95.18 kg^2^), displaying higher dispersion than their direct counterparts. Finally, posterior means for the direct-maternal genetic covariances were negative in both breeds, being the magnitude of the parameter in Angus about half the value obtained for Hereford ( = -27.00 kg vs.  = -56.31 kg). On the contrary, the segregation covariance between direct and maternal genetic effects was positive within the 95% HPD interval. Besides, the posterior mean was  = 9.55 kg^2^ and the posterior mode was 3.20 kg^2^.

Posterior summaries for direct heritability, maternal heritability, and direct-maternal correlation in the reference F_2 _population are presented in Table 4. Heritabilities were defined as the quotient between the additive variance for each trait, computed as the weighted sum of additive variances by source of variability, and the phenotypic variance for the reference breed group. Direct and maternal heritabilities means were 0.27 and 0.18, respectively, with a small shift with respect to the mode in the latter case. In turn, mean direct-maternal correlation was -0.33. The posterior probabilities that all variance quotients are strictly positive were greater than 0.95 in agreement with the 95% HPD intervals.

**Table 4:** Posterior summaries for direct heritability, maternal heritability, and direct-maternal correlation

	Mean (SD)		Mode (LHPD95, UHPD95)	
	
Trait^1^	DWW	MWW	DWW	MWW
**DWW**	0.27 (0.03)	-0.33 (0.13)	0.26 (0.20, 0.33)	-0.35 (-0.57, -0.07)
**MWW**		0.18 (0.03)		0.24 (0.11, 0.24)

^1^DWW = direct weaning weight; MWW = maternal weaning weight; SD = standard deviation; LHPD95, UHPD95 = lower and upper limits for the 95% HPD interval

Heritabilities (diagonals) and correlations (off-diagonals) are expressed with reference to the F_2 _population. Summary measures of heritabilities were calculated using the weighted sum of additive variances by origin divided by the phenotypic variance at each cycle; correlation summaries were computed using the weighted sum of direct-maternal genetic covariance by origin divided by the product of additive standard deviations at each cycle

Finally, relative contributions of each source of variability to the total direct and maternal additive variances in individuals F_2 _are displayed in Table 5. The contribution from the Angus to total direct additive variance was higher than the contribution of Hereford (50.26% vs. 41.73%) while, conversely, Hereford origin accounts for almost twice the maternal additive variance (23.59% vs. 59.65%). In turn, the contribution of the segregation variance to the total additive variance was not significant for the direct component of the trait (< 10%), though it was more important for the maternal component (≈ 17%). However, when the contribution was calculated using the posterior modes, segregation variance contributed in a non-significant fashion in both cases: 3.32% and 5.71% for the direct and maternal components, respectively.

**Table 5:** Direct and maternal additive variances in F2 individuals split by source of variability

		% by source			Total^1^
		
F_2 _individuals additive variances		Angus	Hereford	Segregation	kg^2^
Direct:		50.26%	41.73%	8.01%	120.11
Maternal:		23.59%	59.65%	16.76%	79.78

^1^The total was computed using posterior means

## Discussion

In this study we formalized the equivalence between the multibreed animal model with heterogeneous additive variances introduced by García-Cortés and Toro [10], and the one derived from the quantitative genetic theory [1,2]. In doing so we used a different formulation not including breeding values for the individuals with null contributions within the additive vectors by breed origin. Next we defined appropriate matrices that retrieved the null-row and null-column patterns from the incidence matrices of breeding values and from the partial numerator relationship matrices. Finally, on using these matrices and by means of appropriate algebraic operations, we showed the equivalence between both models. Even though in our derivation we assumed a two-breed composite population, the generalization to *p *breeds requires only redefining the appropriate vectors of breeding values by breed origin.

Further, we extended the model to include maternal effects [2,19] and, in order to estimate (co)variance components, we described a hierarchical Bayes implementation. Generally speaking, the Bayesian approach is more intuitive, more flexible, and its results are more informative when compared to inference methods based on maximizing the likelihood function. The basic idea in the Bayesian approach is to combine the knowledge a priori about the unknown parameters, with the additional information supplied by the data [20]. In particular, within the framework of a multibreed animal model, an advantage of the approach is the possibility to incorporate prior information about the (co)variance components by source of variability [4]. In any case, if there is complete uncertainty about these parameters a priori, a possible action is to consider flat unbounded priors [10]. Alternatively, another option is to use conjugate inverted-gamma distributions as priors, which are parameterized so that they reflect the uncertainty through the degrees of belief chosen by the analyst, as we did in the current application. In both situations, the analytical expression for the full conditional posterior densities is recognizable and, as a consequence, it is possible to implement a Gibbs sampling algorithm as the inference method [37].

In fact, as pointed out by García-Cortés and Toro [10], only a small extra coding effort is required to accommodate a Gibbs sampling algorithm for (co)variance components estimation in the framework of a multibreed animal model with heterogeneous variances. Basically, it is necessary to modify slightly one of the several routines available to compute inbreeding coefficients to appropriately assign contributions to the partial numerator relationship matrices. With this purpose, García-Cortés and Toro [10] used the procedure of Quaas [38]. By contrast, we adapted the subroutine of Meuwissen and Luo [30] as it presents two advantages for the problem at hand: 1) it is a faster algorithm, and 2) it performs on a row by row basis [30,39]. Modifying the Meuwissen and Luo [30] subroutine requires redefining the expression for the within-family variance, and initializing the work variable FI with the appropriate coefficients of breed composition.

Among other important issues, implementing a Gibbs sampler involves choosing a sampling strategy, deciding the number of chains to be generated, and defining the initialization values, length of the burn-in period, and number of cycles needed to ensure a representative sample from the marginal distribution of interest [40]. In this study we used a single-site, systematic scan sampling strategy. For all other issues while implementing the Gibbs sampler, we followed the work of Geyer [33]. Therefore, the results presented here are based on a very long chain after discarding the first 3.4% (100,000) samples as burn-in. The main concern was the extremely high correlations observed between adjacent samples for all (co)variance components. However, it is worthy of note that even though sub-sampling reduced these auto-correlations to reasonable amounts, thinning is not a mandatory practice [41], and certainly is not needed to obtain precise posterior summaries [33].

Another concern is the computing feasibility of the Gibbs sampler described here for large datasets. In this regard, two major issues that affect run-time should be distinguished: first, the number of arithmetic operations needed to accomplish one cycle of the Gibbs sampler as a function of the number of individuals in the pedigree file, and second, the number of cycles necessary to attain convergence. The most time consuming tasks within each round of the procedure are sampling of the location parameter vector, and computing the quadratic forms while sampling the covariance matrices. These steps involve arithmetic operations on the entries of large matrices: the mixed model coefficient matrix and the partial numerator relationship matrices, respectively. Yet, given the sparse storage of these matrices and the fact that arithmetic operations are performed only on non-zero entries, it can be shown that the time per cycle is, ultimately, linear in the number of individuals. It should also be noticed that the system size grows in a quadratic fashion according to the number of breeds involved [10]. However, the increase in the number of equations will be somehow alleviated due to the existence of null equations, and this will depend on the breed composition of the animals in the data file. Now, ascertaining convergence is another issue. In our implementation, formal tests were inconclusive for chain lengths below 1,000,000 cycles for some of the (co)variance components. Particularly, the Raftery and Lewis test computed using the BOA package [34], indicated that there were strong dependencies in the sequences and as a consequence, there was a very slow mixing of the chain. Thus, in a larger data set, strategies to improve the mixing will probably be needed to reduce run-time. A review on such strategies can be found in Gilks and Roberts [42].

The multibreed animal model introduced in the current research was fitted to an experimental Angus × Hereford data set, and for the first time estimates of the full set of genetic (co)variance components described by Cantet and Fernando [2] in a maternal animal model framework were obtained. As a matter of fact, Elzo and Wakeman [11] have reported REML estimates for a multibreed Angus × Brahman herd, but they used a sire-maternal grandsire bivariate model. These authors parameterized the additional variability arising due to differences in allelic frequencies between breeds in terms of the interbreed additive variance [7], a parameter equivalent to twice the segregation variance as defined by Lo et al. [1]. The estimates of the maternal additive interbreed variance and the interbreed additive covariance obtained by Elzo and Wakeman [11] were in absolute terms much greater than the estimates reported here for the equivalent segregation parameters. However, they questioned the validity of those estimates since the number of records they had was small and the number of (co)variance components to be estimated was relatively large. Elzo and Wakeman [11] also indicated that there was very little information on the interbreed parameters contained in their data. In fact, many of the problems associated with small amounts of data spring from difficulties in quantifying properly the estimation error, especially in models with a hierarchical structure [43]. By incorporating uncertainty through probability densities, Bayesian methods overcome this problem [20,43].

We now discuss other issues of the analysis. First, the results obtained in the current research suggest that the allelic frequencies in the two parental breeds that gave rise to the Angus × Hereford population were similar. This is inferred from the almost trivial contribution of the segregation variance to the total additive variance for both the direct and the maternal component of the trait (see [1,3]) when posterior modes are taken as point estimates for the variances. In connection with this, it is worth mentioning that posterior marginal distributions of the segregation (co)variance components were strongly asymmetric, a pattern which has also been reported by Cardoso and Tempelman [4] when analyzing post-weaning data from a Nelore × Hereford crossbred population. In addition, posterior mean values used as point estimates for the direct and maternal heritabilities, and the direct-maternal genetic correlation in the reference population were in agreement with the values found in the literature [44]. It is important to emphasize, however, that under the multibreed animal model presented here, phenotypic variance is specific to each breed composition, so that heritabilities and correlations are meaningful only to each breed group.

Moreover, breed compositions and functions thereof are key features of the multibreed analysis: they are used both for computing the inverses of the partial numerator relationship matrices, as well as regressor variables for fitting breed group and heterosis mean effects. In fact, in order to fit properly the model described here, the breed composition of each individual must be known. However, data sets with precise information on the breed composition of animals are lacking. Also, an adequate data structure is needed in order to obtain accurate estimates of the (co)variance components; for example, only the data from the progeny of crossbred parents provide information to estimate segregation variance [11]. In this respect, the data file used here had exceptional features. First, it contained plenty of interbreed information, with records collected on individuals pertaining to several breed groups, and with many pedigree relationships connecting groups to each other. In addition, it had a suitable data structure to estimate (co)variance components from maternal animal models [45,46]: a high percentage of the dams had their own records, and a high proportion of the cows had more than one calf. It would be interesting to assess the performance of the multibreed analysis described here with field data, especially when interbreed information is limited.

## Conclusions

Theoretical and empirical considerations justify the use of a heterogeneous genetic covariance structure when fitting multibreed animal models. In this regard, the approach based on the decomposition of the genetic covariance matrix by source of variability [10] simplifies the problem of estimating the (co)variance components by using a Gibbs sampler. In fact, our results show that the ensuing model is equivalent to the one described in [2]. Furthermore, the extension to include maternal effects and the implementation of the hierarchical Bayes analysis is straightforward. Additionally, we fitted weaning weight data from an experimental Angus × Hereford population, and we obtained, for the first time, estimates of the full set of genetic (co)variance components, including a positive estimate for the direct-maternal segregation covariance.

## Competing interests

The authors declare that they have no competing interests.

## Authors' contributions

SML conceived, carried out the study and wrote the manuscript; RJCC conceived and supervised the study. Both authors read and approved the final manuscript.

## Appendix

### Full conditional posterior densities

Starting from the joint posterior distribution in (17), it is possible to identify the full conditional posterior density of any parameter of interest by keeping the rest of them fixed. In this section we will present the analytic expression for the full conditional densities arising from the multibreed maternal animal model introduced in (10). Detailed derivations can be found in Sorensen and Gianola [20], and Jensen et al. [24].

Let the location parameter vector ***θ*** be such that . The full conditional distribution of this vector is then proportional to

(A1)

Explicitly, (A1) is equal to

(A2)

Now, by means of appropriate algebraic operations it can be shown [24] that

(A3)

Here,  is the solution to the mixed models equations arising from model (10), ***C***^-1^ is the corresponding inverse coefficient matrix, and ***r*** the right hand side. Unlike the mixed model equations presented by García-Cortés and Toro [10], the system derived from (10) has a unique solution. It should be reminded that under this formulation, it is necessary to add  to the diagonal entry corresponding to every fixed effect, where *k*_*i*_ reflects a prior state of uncertainty about the location parameters.

Next, we focus on the full conditional posterior distribution of the error variance. This distribution is proportional to

(A4)

and explicitly equals to

(A5)

Define then

(A6)

Hence, it is verifiable that

(A7)

An inspection of expression (A7) reveals that this is the kernel of a scaled inverted Chi-square density with parameters  and . In short

(A8)

Next, note that the full conditional posterior distribution of the genetic covariance matrix by source of variability *X *(*X* = {*A*, *B*, *S*}) is proportional to

(A9)

In (A9), the symbol ***G***_0*R*_ is used to represent the genetic covariance matrices for the other sources of variability. Under the conditional distribution of ***G***_0*X*_, these matrices are taken as constants. Then, according to (24), conditional distribution (A9) can be written explicitly as

(A10)

The last expression is recognizable as the kernel of the Inverted Wishart distribution . A similar result can be used to obtain the full conditional distributions of the two other genetic covariance matrices by source of variability.

Finally, it remains to specify the full conditional posterior distribution of the maternal permanent environmental variance. This density is proportional to

(A11)

and explicitly to

(A12)

On defining

(A13)

it is verifiable that

(A14)

It follows by inspection that density (A14) is in the form of a scaled inverted Chi-square density with parameters  and , so that

(A15)

## References

[B1] Lo LL Fernando RL Grossman M Covariance between relatives in multibreed populations: additive model Theor Appl Genet 1993 87 423 430 10.1007/BF00215087 24190314

[B2] Cantet RJC Fernando RL Prediction of breeding values with additive animal models for crosses from two populations Genet Sel Evol 1995 27 323 334 10.1186/1297-9686-27-4-323

[B3] Birchmeier AN Cantet RJC Fernando RL Morris CA Holgado F Jara A Santos Cristal M Estimation of segregation variance for birth weight in beef cattle Livest Prod Sci 2002 76 27 35 10.1016/S0301-6226(02)00013-1

[B4] Cardoso FF Tempelman RJ Hierarchical Bayes multiple-breed inference with an application to genetic evaluation of a Nelore-Hereford population J Anim Sci 2004 82 1589 1601 15216984 10.2527/2004.8261589x

[B5] Elzo MA Famula TR Multi-breed sire evaluation procedures within a country J Anim Sci 1985 60 942 952

[B6] Elzo MA Recursive procedures to compute the inverse of multiple trait additive genetic covariance matrix in inbreed and noninbreed multibreed populations J Anim Sci 1990 68 1215 1228

[B7] Elzo MA Restricted maximum likelihood procedures for the estimation of additive and nonadditive genetic variances and covariances in multibreed populations J Anim Sci 1994 72 3055 3065 7759353 10.2527/1994.72123055x

[B8] Vergara OD Ceron-Muñoz MF Arboleda EM Orozco Y Ossa GA Direct genetic, maternal genetic, and heterozygosity effects on weaning weight in a Colombian multibreed beef cattle population J Anim Sci 2009 87 516 521 10.2527/jas.2007-0636 18952738

[B9] Vergara OD Elzo MA Ceron-Muñoz MF Arboleda EM Weaning weight and post-weaning gain genetic parameters and genetic trends in a Blanco Orejinegro-Romosinuano-Angus-Zebu multibreed cattle population in Colombia Livest Sci 2009 124 156 162 10.1016/j.livsci.2009.01.008

[B10] García-Cortés LA Toro MA Multibreed analysis by splitting the breeding values Genet Sel Evol 2006 38 601 615 17129562 10.1186/1297-9686-38-6-601 PMC2689266

[B11] Elzo MA Wakeman DL Covariance components and prediction for additive and nonadditive preweaning growth genetic effects in an Angus-Brahman multibreed herd J Anim Sci 1998 76 1290 1302 9621935 10.2527/1998.7651290x

[B12] Wright S Evolution and the genetics of populations Genetics and biometrical foundations 1968 1 Chicago: University of Chicago Press

[B13] Lande R The minimum number of genes contributing to quantitative variation between and within populations Genetics 1981 99 541 553 7343418 10.1093/genetics/99.3-4.541 PMC1214520

[B14] Emik LO Terril CE Systematic procedures for calculating inbreeding coefficients J Hered 1949 40 51 55 18116093 10.1093/oxfordjournals.jhered.a105986

[B15] Henderson CR A simple method for computing the inverse of a numerator relationship matrix used in prediction of breeding values Biometrics 1976 32 69 83 10.2307/2529339

[B16] Quaas RL Additive genetic model with groups and relationships J Dairy Sci 1988 71 1338 1345 10.3168/jds.S0022-0302(88)79691-5

[B17] Henderson CR Equivalent linear models to reduce computations J Dairy Sci 1985 68 2267 2277 10.3168/jds.S0022-0302(85)81099-7

[B18] Henderson CR Estimation of genetic parameters (abstract) Ann Math Statist 1950 21 309 310

[B19] Willham RL The covariance between relatives for characters composed of components contributed by related individuals Biometrics 1963 19 18 27 10.2307/2527570

[B20] Sorensen D Gianola D Likelihood, Bayesian, and MCMC methods in quantitative genetics 2002 NY: Springer-Verlag

[B21] Hobert JP Casella G The effects of improper priors on Gibbs sampling in hierarchical linear models J Amer Statist Assoc 1996 91 1461 1473 10.2307/2291572

[B22] Cantet RJC Birchmeier AN Steibel JP Full conjugate analysis of normal multiple traits with missing records using a generalized inverted Wishart distribution Genet Sel Evol 2004 36 49 64 10.1186/1297-9686-36-1-49 14713409 PMC2697179

[B23] Wang CS Rutledge JJ Gianola D Marginal inferences about variance components in a mixed linear model using Gibbs sampling Genet Sel Evol 1993 25 41 62 10.1186/1297-9686-25-1-41

[B24] Jensen J Wang CS Sorensen DA Gianola D Bayesian inference on variance and covariance components for traits influenced by maternal and direct genetic effects, using the Gibss sampler Acta Agric Scand 1994 44 193 201

[B25] Morris CA Baker RL Cullen NG Johnson DL Rotation crosses and *inter se *matings with Angus and Hereford cattle for five generations Livest Prod Sci 1994 39 157 172 10.1016/0301-6226(94)90181-3

[B26] Hill WG Dominance and epistasis as components of heterosis J Anim Breed Genet 1982 99 161 168

[B27] Lynch M Walsh B Genetics and analysis of quantitative characters 1998 Sunderland, MA: Sinauer Associates

[B28] Misztal I Computational techniques in animal breeding. Course notes http://nce.ads.uga.edu/~ignacy

[B29] Misztal I Tsuruta S Strabel T Auvray B Druet T Lee DH BLUPF90 and related programs (BGF90) 7th World Congress on Genetics Applied to Livestock Production: 19-23 August 2002; Montpellier 2002

[B30] Meuwissen THE Luo Z Computing inbreeding coefficients in large populations Genet Sel Evol 1992 24 305 313 10.1186/1297-9686-24-4-305

[B31] Quaas RL Pollak EJ Mixed model methodology for farm and ranch beef cattle testing programs J Anim Sci 1980 51 1277 1287

[B32] Gilmour AR Gogel BJ Cullis BR Thompson R ASReml User Guide Release 2.0 2006 Hemel Hempstead, HP1 1ES, UK: VSN International Ltd

[B33] Geyer CJ Practical Markov chain Montecarlo Stat Sci 1992 7 473 511 10.1214/ss/1177011137

[B34] Smith B boa: An R package for MCMC output convergence assessment and posterior inference J Stat Soft 2007 21 1 37

[B35] The R Project for Statistical Computing http://www.r-project.org/

[B36] Silverman BW Density estimation for statistics and data analysis 1986 London: Chapman and Hall

[B37] Gelfand AE Smith AFM Sampling-based approaches to calculating marginal densities J Am Stat Assoc 1990 85 398 409 10.2307/2289776

[B38] Quaas RL Computing the diagonal elements and inverse of a large numerator relationship matrix Biometrics 1976 32 949 956 10.2307/2529279

[B39] Mrode RA Linear models for the prediction of animal breeding values 2005 Wallingford, Oxfordshire, UK: CAB International

[B40] Gilks WR Richardson S Spiegelhalter DJ Markov chain Monte Carlo in practice 1996 Boca Raton, US: Chapman and Hall

[B41] Raftery AE Lewis SM Gilks WR, Richardson S, Spiegelhalter DJ Implementing MCMC Markov chain Monte Carlo in practice 1996 Boca Raton, US: Chapman and Hall 115 130

[B42] Gilks WR Roberts GO Gilks WR, Richardson S, Spiegelhalter DJ Strategies for improving MCMC Markov chain Monte Carlo in practice 1996 Boca Raton, US: Chapman and Hall 89 114

[B43] O'Hara RB Cano JM Ovaskainen O Teplitsky C Alho JS Bayesian approaches in evolutionary quantitative genetics J Evol Biol 2008 21 949 957 10.1111/j.1420-9101.2008.01529.x 18373658

[B44] AAABG Genetic Parameters http://www.gparm.csiro.au/index.html

[B45] Gerstmayr S Impact of data structure on the reliability of the estimated genetic parameters in an animal model with maternal effects J Anim Breed Genet 1992 109 321 336

[B46] Maniatis N Pollot G The impact of data structure on genetic (co)variance components of early growth in sheep, estimated using an animal model with maternal effects J Anim Sci 2003 81 101 108 12597378 10.2527/2003.811101x

